# Supplementary material for: Complexome profiling of mitochondria in the myzozoan parasite of oysters, Perkinsus marinus
Source: PLoS Pathog. 2026 Sep 29;22(9):e1014543. doi: 10.1371/journal.ppat.1014543 (PMC13623125; doi:10.1371/journal.ppat.1014543)
Supplement: Supplementary file 1 [file ppat.1014543.s015.pdf]

Fig. 2A

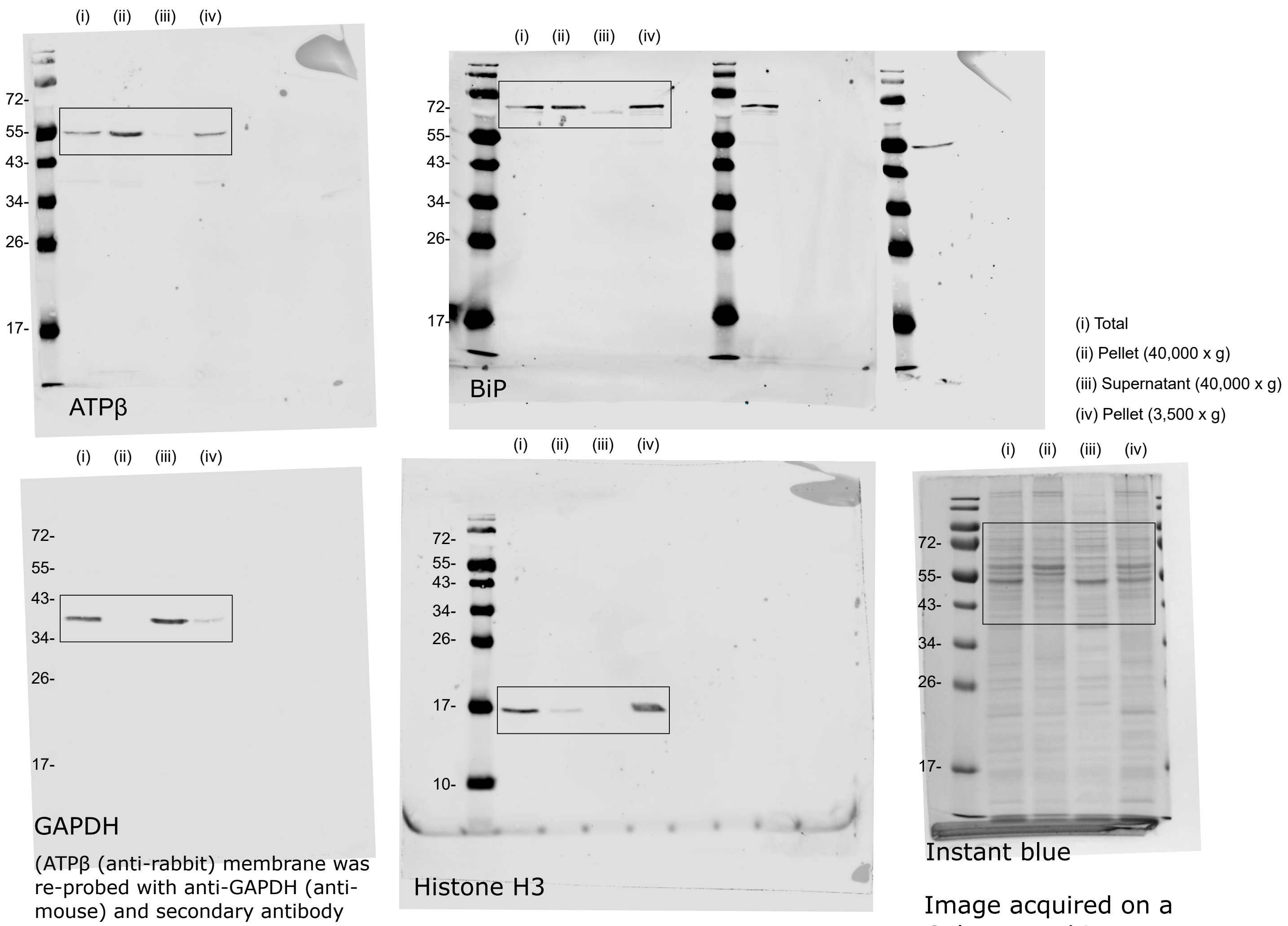

(ATPβ (anti-rabbit) membrane was re-probed with anti-GAPDH (anti-mouse) and secondary antibody fluorescence signal acquired on a seperate channel)

Image acquired on a GelDoc machine.

Images acquired on an Odyssey CLx machine.

Fig. 2B

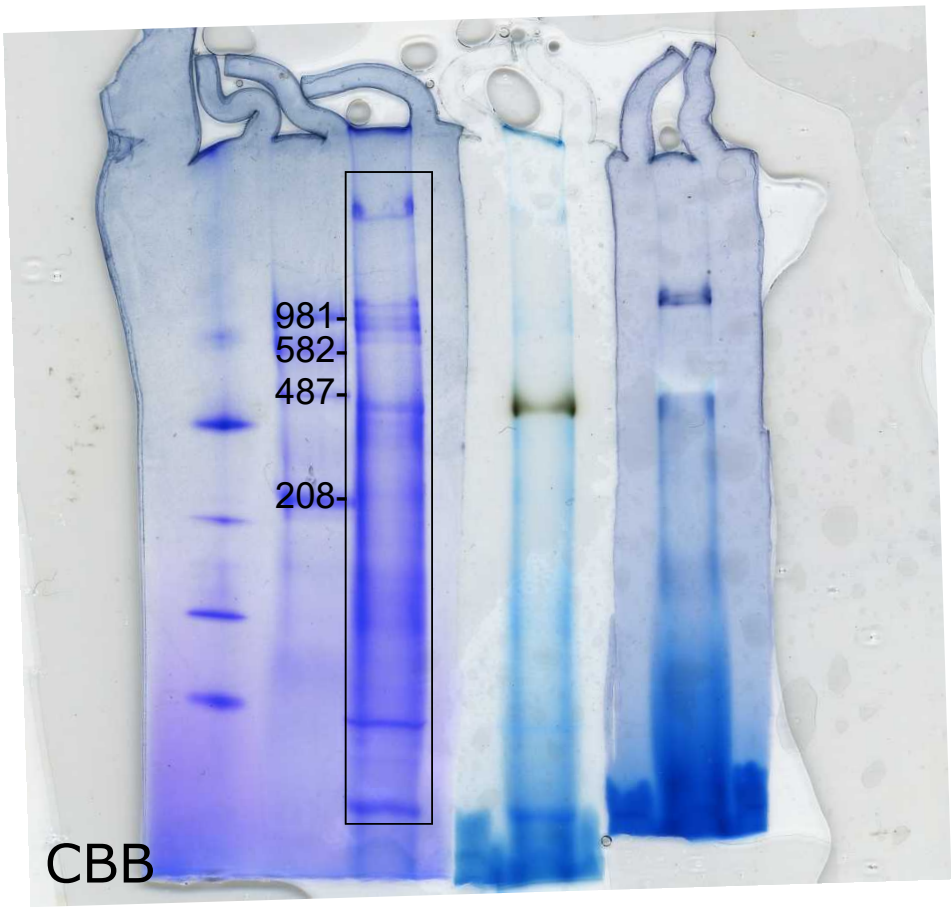

Image acquired on an Epson scanner.

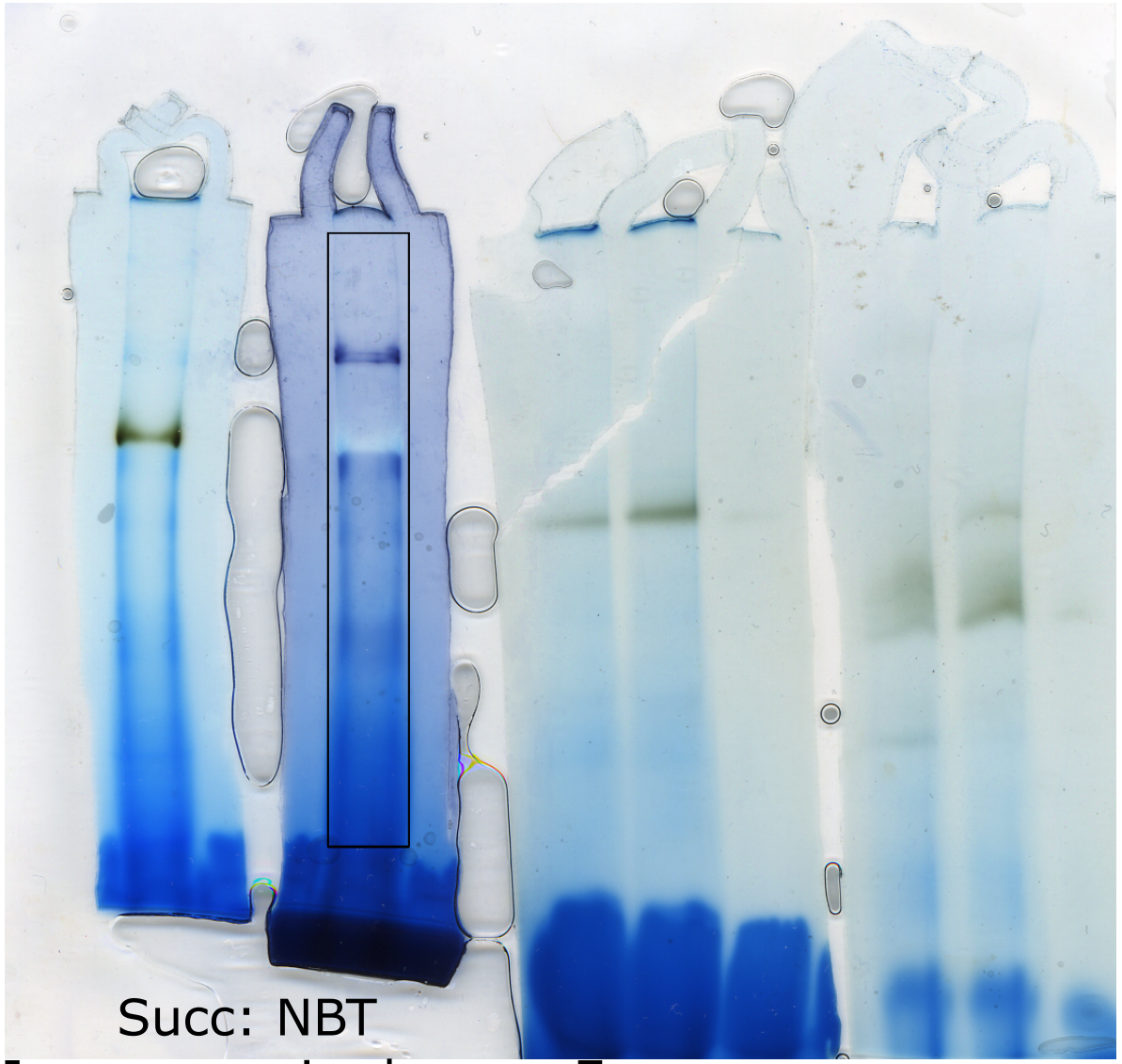

Image acquired on an Epson scanner.

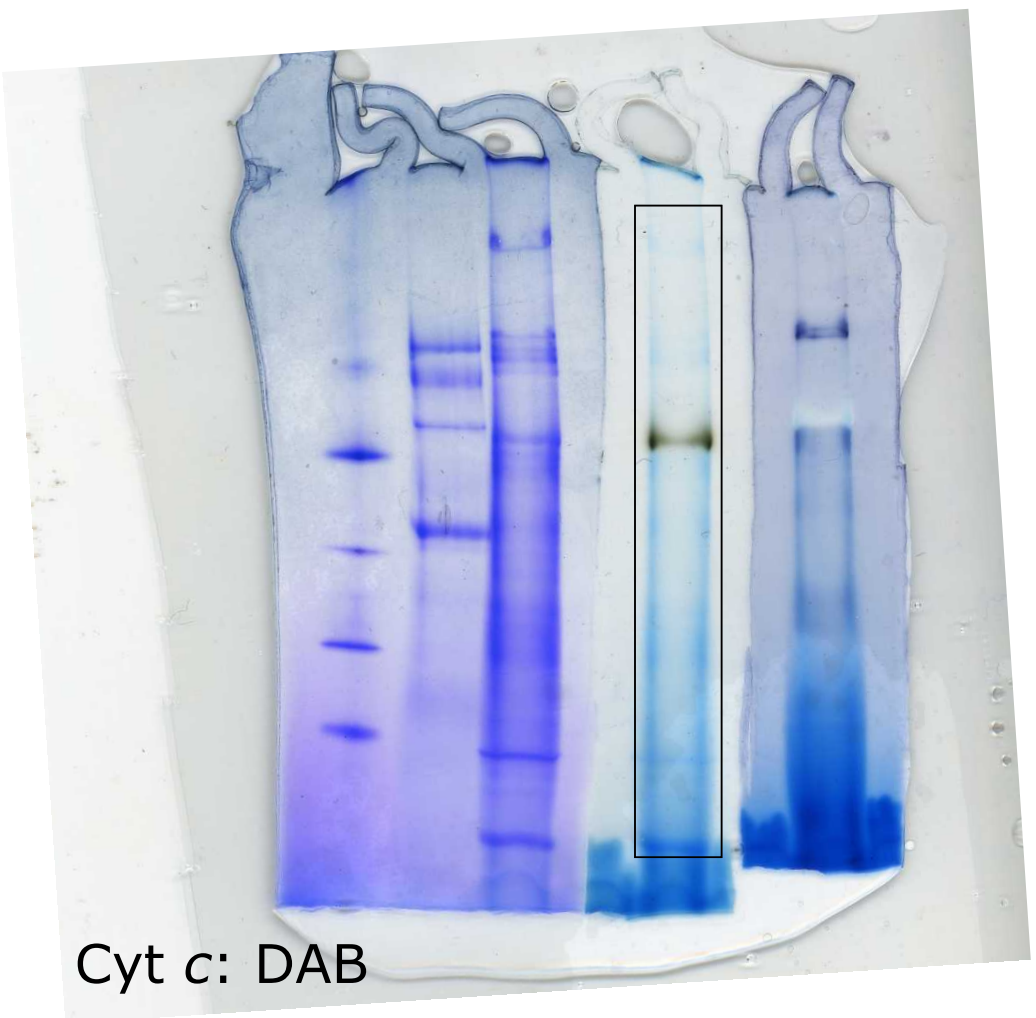

Image acquired on an Epson scanner.

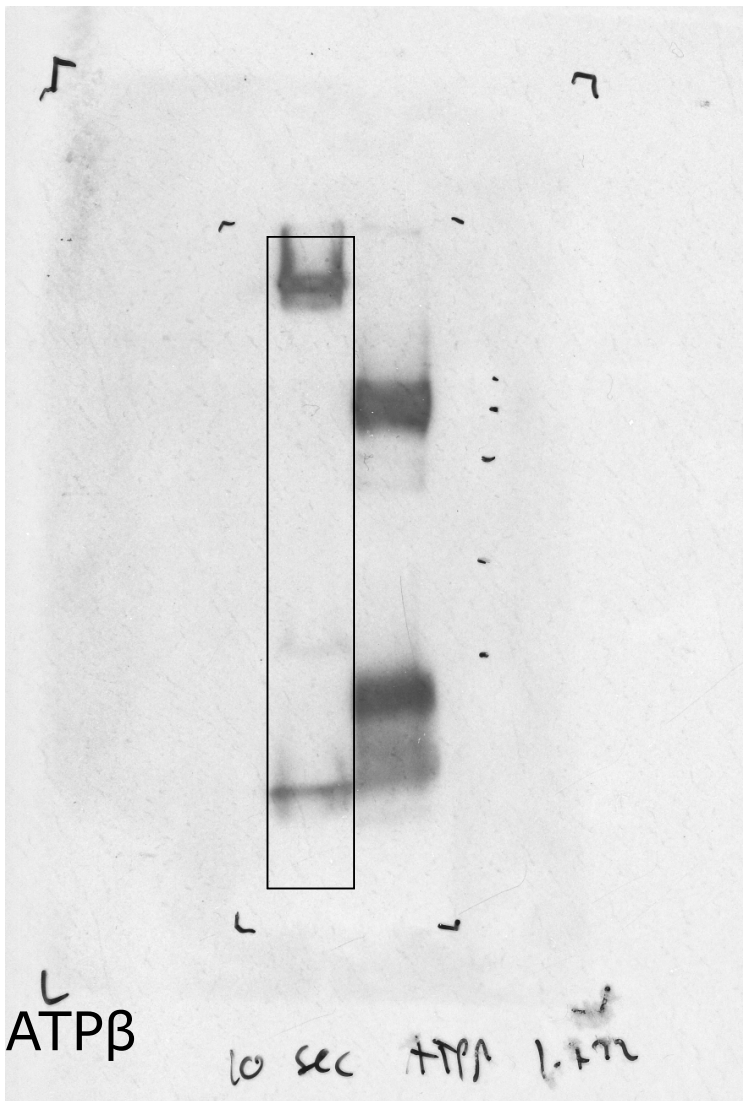

Image acquired on an x-ray film developer, follwed by an Epson scanner.

S1 Fig.

Replicate 1

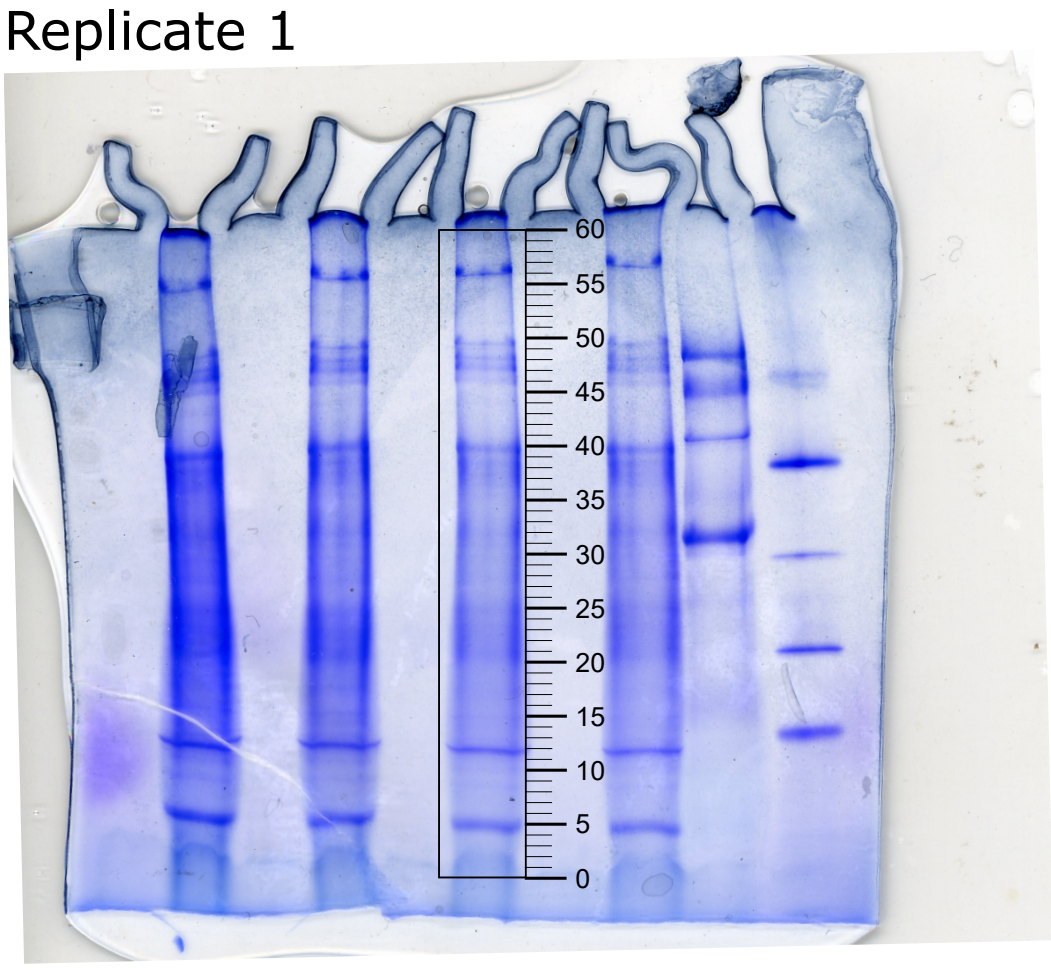

Image additionally used in Fig 2C, 3A, 4, 5A, 5B.

Replicate 2

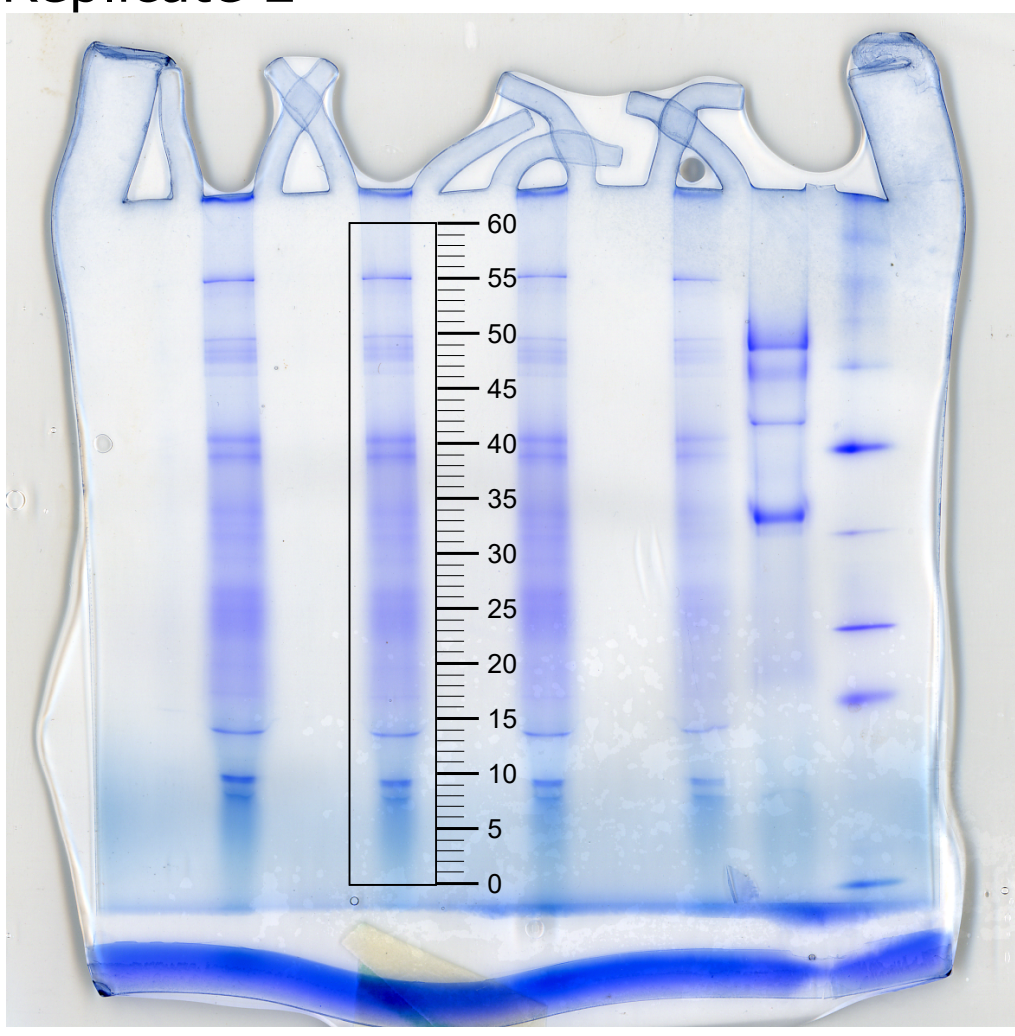

Image additionally used in Fig 5C.

Whole cell

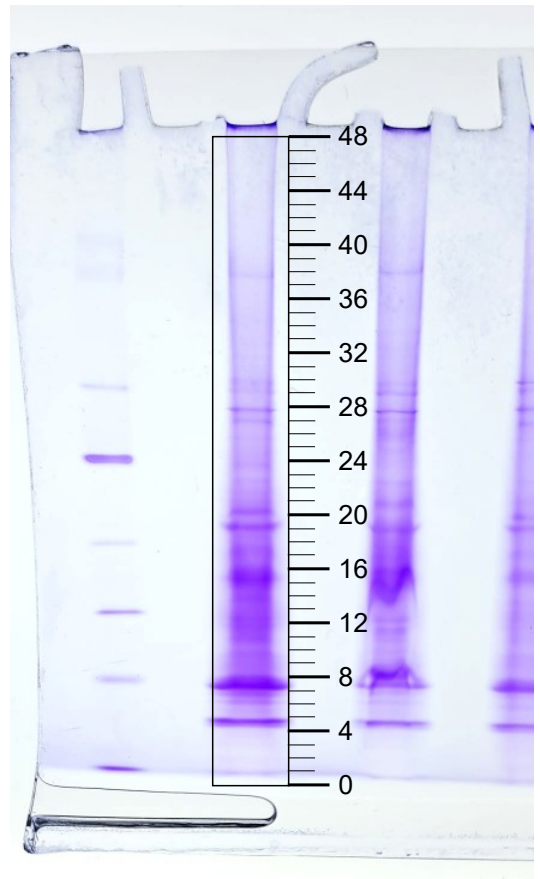

Image additionally used in Fig S2C, S3A, S5B.

Images acquired on an Epson scanner.
