# Supplementary material for: Complexome profiling of mitochondria in the myzozoan parasite of oysters, Perkinsus marinus
Source: PLoS Pathog. 2026 Sep 29;22(9):e1014543. doi: 10.1371/journal.ppat.1014543 (PMC13623125; doi:10.1371/journal.ppat.1014543)
Supplement: Supplementary file 2 [file ppat.1014543.s017.docx]

Response to reviewers

*Dear editor,*

*We would like to thank you and the reviewers for taking the time to review our manuscript, and for the thorough and thoughtful comments that have helped us improve it. We are pleased that all reviewers highlighted the quality of the work, the well-supported conclusions and importance of exploring mitochondrial biology in previously understudied unicellular pathogens. All reviewers provide helpful suggestions for improvement, predominantly along the lines of setting our findings in a broader evolutionary context by performing comparative analysis across various lineages of the myzozoans.*

*Below, in blue and italicised text, is a point-by-point response to the suggestions. We addressed almost all the comments with a revision to the manuscript and where possible with new work as requested.*

*In the “Revised manuscript with track changes” document, deletions are denoted by the track change function and additions by yellow highlighted text. Please note, the line numbers we refer to below correspond to the line numbers in the unmarked “Maclean_Manuscript” document.*

*We feel our changes, as directed by reviewer suggestions, greatly strengthen the manuscript and put our findings in a broader comparative context.*

**Major:**

Reviewer 1:

1. A major conclusion of the study is that a lot of the unique features and proteins of apicomplexan mitochondrial complexes are conserved in Perkinsus. This provides evidence that these novelties were already present in the common ancestor of myzozoans. However, the study makes only a limited attempt at describing these orthologies, and buries a lot of it in the supplementary data (e.g. Table S4 and the ATPTG1 homology data). The study would be improved by providing a more thorough (and visual) analysis of which proteins identified in Perkinsus complexes have homologs in apicomplexans, and which of these proteins are present in the characterized complexes of apicomplexans. The authors might also consider providing a more detailed analysis of which Perkinsus proteins do NOT have homologues in apicomplexans (and vice versa), since this may point to lineage-specific novelties within the myzozoans (as they do for ATP synthase in Figure 3C). Additionally, it would be interesting to note which "novel" myzozoan mitochondrial complex *proteins also have homologs in ciliates, given that ciliates are the sister taxon to the myzozoans.*

*We thank reviewer 1 for this suggestion. We have undertaken significant extra analysis to assess the conservation of mETC subunits across different myzozoan and ciliate lineages. In particular, we took all mETC subunits identified in our complexome and the recent Perkinsus mETC structure as a starting point and performed sequence-based homology searches (using both BLAST and HMM tools) across the myzozoans and also some ciliate species. We covered dinoflagellates, apicomplexans and chrompodellids, as well as species which only possess a subset of complexes (for example Chromera velia has lost complex III, and Cryptosporidium muris possesses complex II, but not complex III and IV). The latter acts as a control to show that the pattern of loss and retention of complexes matches the loss and retention of subunits we are searching.*

*These new analyses now form new panels in main figure 2 (D) and 3 (B), displaying the data as retention and loss of individual subunits in a Coulson diagram. We also included the corresponding full dataset in newly supplemental table S6. We also added a substantial new text to the relevant results section (lines 225-228; 262-274; 289-291; 334-335) discussing this new analysis, highlighting conservation across the myzozoans and making a comparison with the ciliates.*

Reviewer 2:

1. The current figures that predominantly show the BN-PAGE slices are not so helpful or informative in summarizing the key proteomic findings. Summary diagrams should be included as a panel within each figure to schematically depict the protein subunits of the studied complexes, similar to schematic figures included in the authors’ prior complexome studies of Toxoplasma (or something similar to Fig. 2 in PMID 23371551). Summary panels that list the identified subunits (perhaps in comparison to Toxoplasma and/or Plasmodium) should also be considered. If necessary, some of the slice details in the present figures can be decanted to supporting figures.

*We appreciate our initial figures lent heavily on heatmaps, which is the traditional way to display complexome data, but this lacks visual appeal and may not be the most informative way to convey findings. To remedy this, we have done the following:*

- *Multiple heatmaps have been moved to supplemental figures (S2A, S3A, S5B).*
- *We have added new diagrams (in response to both reviewer 1 and 2) in figure 2D and 3B which display the composition of each of the mETC complexes, comparing them to the well-studied Plasmodium and Toxoplasma complexes as well as performing homology searches on key species across various ciliate and myzozoan lineages.*
- *We have added a new figure 1A to display the mETC and ATP synthase in humans and Perkinsus, along with the subunit number found in each complex, and a comparison to the well characterised apicomplexan Toxoplasma gondii, summarising the findings of our paper.*

2. Evolutionary and phylogenetic comparisons are central to the major conclusions of this manuscript. It would be helpful to include a simple phylogenetic tree as a new Figure 1, showing the relationships between Perkinsus, Apicomplexa (including Toxo and Plasmodium that are key comparison organisms), and other major eukaryotic clades (including opisthokonts).

*We have created a new figure 1. Panel A (also discussed above) is a schematic depiction of the complexes, and their subunit composition in Perkinsus and humans, which possess one of the best characterised respiratory chains and is an important comparison throughout the study. For panel B we have made a schematic which shows the relationship between the main lineages of the myzozoans. Included in this diagram are species used in the new homology analyses done in figure 2 and 3, as well as the composition of their respective respiratory chains.*

Reviewer 3:

Line 49: Do we know which subunits of the mETC complexes are encoded in the mitochondrial genome of P. marinus, and are those the same as the ones in apicomplexans? If so, the authors should mention it.

*The Perkinsus marinus mitogenome has recently been sequenced (PMID: 36108082) and encodes complex III subunit cytochrome b and the complex IV subunits coxI and coxIII. We have added text to explicitly mention this fact in lines 238-239.*

Line 110: The last sentence of this paragraph is somewhat misleading.
Although the authors convincingly show that their mitochondrially enriched fractions are indeed enriched with mitochondria, the ER is also present. Please mention that.

*We have reworded the sentence to read “*These data suggest that the purification protocol was able to obtain material enriched in **organellar** proteins*” (line 124-125) to better reflect the fact this fraction is enriched in both mitochondrial and ER proteins, as discussed in the preceding sentences.*

On figure 2E, What is the degree of confidence in the alphafold predictions of ISPR? This is also valid for the other proteins shown in the paper.

*We have added each prediction’s pTM (predicted template modelling) score to the figure legend. The pTM score is a measure of the accuracy of the entire structure, with a standard cut-off set at 0.5 (i.e. scores above 0.5 are considered a reasonably good prediction). In addition, images of alphafold predictions have been recoloured to reflect the confidence level for each region of the protein (coloured according to pLDDT score). Predictions of new subunits ATPPM1 and 2 have been removed, as we feel these were not informative.*

Line 167: When the authors are comparing their mitochondrial datasets with published ones from Toxoplasma and Plasmodium, what determines a “sufficient” coverage? Is there a certain percentage that needs to be attained? Is this arbitrary? The authors should explain their reasoning.

*We have modified the text to read “*This includes 67-70% of proteins belonging to the “mitochondrial membranes” cluster, **a comparable proportion to that obtained in our previous complexome study of Toxoplasma mitochondrial complexes*”*** (lines 177-179) *and “*Overall, these comparisons suggest our complexome profiles **have similar** coverage of mitochondrial proteins **with previous complexome** studies*” (lines 187-188). We also removed the text implying there was a particular arbitrary threshold that needed to be crossed to perform further analysis. We feel this change retains the informative comparison of the coverage obtained in the two studies, while removing the impression of an arbitrary threshold that needs to be met.*

Line 210: has the homolog of BCS1 been observed in the author’s published T. gondii complexome analysis? If so they should mention it, it not they should also mention it and explain why is that.

*Based upon comments from both this reviewer and reviewer 1 (below in minor comments) we have removed the section referring to BCS1 as it is speculative.*

Line 305: Since a putative complex III assembly factor was detected, could those proteins be ATP synthase assembly factors instead of subunits of the complex? Is there a way to distinguish those using the author’s approach?

*We agree that it is possible that these new subunits could also be late-stage assembly factors, and that it is not possible to distinguish between the two possibilities with our data. We have modified the text to reflect this (lines 360-361).*

Line 413-414: Is there any relevance of detecting proteasome proteins in the mitochondrially enriched fractions? Could this be a contamination? The authors did not mention this in the manuscript (Table S5).

*We agree that the detection of the proteosome suggests contamination of the organellar fraction with other cell compartments. We have added text to highlight this (lines 483-488).*

Why did the authors used BDDM instead of digitonin as they have previously reported (PMID: 33651838)? In BN-PAGE, digitonin seem to reveal complex arrangements that are lost with BDDM. A comparison of using both detergents will be useful.

*Both the current work and our previous Toxoplasma work used βDDM for the complexome study. As both studies were the first of their kind in Toxoplasma and Perkinsus, our main interest was to determine the composition of individual complexes. While digitonin maintains intra-complex associations, and is therefore useful for exploring how complexes interact with one another, βDDM-derived complexome profiling allows for simpler analysis of the composition of individual complexes. This is because using βDDM complexes typically resolve as a single clear band in the gel, allowing for easier assignment of co-migrating proteins, whereas using digitonin complexes may have multiple peaks, confounding the assignment of novel proteins to the complex.*

*In the current work we further added the whole cell complexome, performed using the milder detergent IGEPAL, and this allowed determination of the interactions between complexes. Our study therefore used both a mild and a harsher detergent, allowing detailed determination of complex composition as well as the interactions between complexes.*

**Minor:**

Reviewer 1:

2. Line 75. “While apicomplexan mitochondrial genomes encode the lowest number of protein-coding genes known [20], their ribosomal RNAs (rRNAs) are uniquely extremely fragmented [21,22].” True that mitochondrial rRNAs are extremely fragmented in apicomplexans, but this isn’t entirely unique (e.g. also occurs in dinoflagellates and some ciliates). Additionally, Chromera velia encodes two protein-coding genes in its mitochondrial genome, which is fewer than in apicomplexans. Consider re-wording for accuracy.

*We have amended the text to remove the word “unique” and removed the incorrect assertion that apicomplexans have the lowest number of mitochondrial genes (line 91).*

3. Line 173. “recent studies in apicomplexans suggest a much-expanded subunit repertoire compared to the commonly studied opisthokont models”. Given that opisthokont protein complex compositions provide an important comparison to the myzozoan complexes throughout the manuscript, consider briefly mentioning here which lineages belong to the opisthokonts.

*We have reworded this section for clarity, explicitly mentioning mammalian and yeast models (line 193). Throughout the manuscript we have replaced references to the opisthokonts with mammals and yeast.*

4. Line 194. “This size of complex II is consistent with the BN-PAGE in-gel stain (Fig 1B).” Given that the in-gel stain depends on enzymatic activity, it might be worth pointing out this size is therefore likely the enzymatically active version of the complex.

*We have added this point to the text (line 213-214).*

5. Line 210. “we observed Pmar_PMAR021073, a predicted homolog of the complex III assembly factor BCS1, with a peak in the gel slice below mature complex III, at ~820 kDa … suggesting that in our mitochondrially-enriched complexome, we observe a late assembly intermediate as well as mature complex III.” This claim seems a bit speculative, since there are other proteins in the same ~820 kDa peak that are presumably not part of any assembly intermediate.

*We agree that this is a speculative statement, and have removed it from the text.*

6. Line 233. “supercomplexes” (spelling)

*We have corrected this in the text.*

7. Line 240. “complex IV subunits have peaks at ~760-840, ~1,300-1,400 and ~2,800 kDa”. How were these mass estimates determined? If none of the calibration standards are above 2,800 kDa, then probably better to describe this without putting a number on the mass.

*We have removed this from the text and made our point without using numbers.*

8. Line 245. “demonstrating that the enlarged mETC complexes observed in Apicomplexa are conserved across a broader group within the myzozoans and thus likely evolved in their common ancestor.” The masses of the complexes being enlarged is not, in itself, strong evidence that complex enlargement occurred in the common ancestor of myzozoans. Rather, the presence of myzozoan lineage-specific subunits (which are contributing to the larger masses) suggest that the novelty observed in myzozoan mitochondrial complexes was already present in the common ancestor of the lineage. A more thorough comparison of orthology in mitochondrial complex proteins across myzozoans may be a more robust way to phrase this argument (see Major comment 1).

*We have amended the text which now reads “*Our complexome datasets allow identification of almost complete mETC complexes, validating their composition. Sequence-based homology searches suggest the composition of mETC complexes is broadly conserved across the myzozoans but not in the ciliate lineage*.” (lines 288-291) This states the conclusions of this section, and we have moved further interpretation of these data to the discussion section.*

9. Line 312. “Homology analysis showed that this fusion was conserved across a subset of apicomplexans and that outside this group ATPTG1 and CytC1 are encoded by separate genes [10] (S3C, D, S4 Figs, Table S7).” The authors could be clearer that they encoded on separate genes in Perkinsus. Similarly, in the next line, the authors could be clearer that they are referring to the P. marinus homolog of ATPTG10: “Our detection of ATPTG1 in complex with ATP synthase [in P. marinus] strongly suggests …”

*We have amended text for clarity (lines 369, 372).*

10. Table S4. Consider adding a comparison to additional apicomplexan species (especially Plasmodium falciparum, given that complexome data exist for that organism), and also listing VEuPathDB gene IDs for the equivalent apicomplexan homologs. See also Major comment 1 – consider providing a more visual representation of these data, and including at least some of the analysis in the main figures.

*In the course of our additional analysis we have significantly revised and updated table S4 to include information about corresponding subunits in Toxoplasma and Plasmodium which are experimentally validated, and added a new table (S6). Such data is also visually represented in figures 2D and 3B.*

11. Line 349. “11 Toxoplasma proteins interact each with multiple rRNAs and therefore were assigned a putative role as “joints”.” Joints in what sense? That they are joining different rRNA fragments? This could be clearer.

*We have reworded for clarity (lines 405-409).*

12. Line 400. “Further investigations will be required to identify if additional TOM subunits exist, in particular divergent versions of the receptors Tom20 and Tom70.” Structural analyses of the TOM complex of opisthokonts have characterized a so-called “core” TOM complex, consisting of Tom40/22/7/6/5 (e.g. PMIDs 31740857 and 33083003). This suggests that the receptor proteins of this complex (e.g. Tom70/20 in opisthokonts) may associate only transiently with the complex. The identification of candidate Tom5 and Tom6 homologs in Perkinsus suggest that the entire core TOM complex must have evolved very early in eukaryotic evolution, which is something the authors could consider highlighting. I was curious whether the candidate Perkinsus Tom5/6 homologs are present in apicomplexans as well? This may be worth commenting on here.

*Homology searches suggest one of these new putative TOM subunits is conserved in the apicomplexan species Toxoplasma gondii and Plasmodium falciparum, but not in other myzozoans or ciliates. In our previous complexome a small protein, TGGT1_288245, with homology to HsTom5 was found co-migrating with Tom40, but there is not a clear homolog of this protein in Perkinsus. This shows how challenging it can be to provide strong evidence of homology for very short proteins. However, it does suggest that both apicomplexans and Perkinsus possess a complete core TOM complex and that this likely evolved early in eukaryotic evolution. We have updated the text to discuss this (437-443, 457-467).*

13. Line 415. “we searched our mitochondrial complexome for the endoplasmic reticulum membrane protein complex (EMC).” What is the role of the EMC in ER biology? What is known about its presence in different eukaryotic lineages?

*We have rectified this by adding text to this section of the results (lines 491-503).*

14. Line 448. “… findings have been found …” Consider re-wording for clarity.

*We have reworded for clarity.*

15. Figure 1 legend. Define “CBB”

*We have updated the text.*

Reviewer 2:

1. The current title seems redundant. The authors may wish to consider a revised title such as “Molecular profiling of mitochondrial complexes in…”.

*The authors collectively agree that the current title is suitable, and thus we chose to keep it. The redundant part is meant for general audiences that may use additional information about Perkinsus.*

2. L81-83: Can the authors elaborate on this point? How many of the identified subunits of the studied complexes were predictable by sequence homology? How many would not have been predictable from sequence comparisons alone? The authors invested substantial effort to experimentally identify these subunits. It would be helpful in the Discussion to comment on the role and impact of experiment in an era in which sequence comparisons (now boosted by the ability to perform structure-based similarity searches as well) are increasingly facile and powerful.

*We have added a substantial section to the discussion to address this (lines 549-577), and discussed the added value that direct laboratory-based experiments such as proteomics or structural projects have compared to sequence-based homology searches alone, especially when non-common model organism sequences are involved. We highlight from our data examples where we have identified subunits, for example ATP synthase subunits ATPPM1, ATPPM2 and SDHC, which would not have been uncovered from homology searches alone, and pointed out the insights gained in the case of ATPTG1, which would not have been apparent from homology searches alone. In total 9 subunits seen in Perkinsus would not have been detected by homology searches. In truth, no one set of techniques gives a full picture of complex composition across a phylogenetically diverse range of species. A careful combination of insights from sequence-based homology searches, and targeted experiments provides the fullest picture of complex diversity in the myzozoans.*

3. Related, several of the authors of the present manuscript are also listed as authors on a preprint structural study of Perkinsus mitochondrial supercomplexes available at bioRxiv. A more detailed comparison of the compositions of mitochondrial complexes determined by mass spectrometry in this study versus direct structural visualization in the parallel study would be very helpful, either within Results or a summary Discussion. The present study reported analysis of additional complexes not observed in the structural paper, but comparison between those complexes in common between the two studies would be appropriate.

*We have added text in the results (line 222, 259-261), expanding our original comparison to the structure in the initial submission, and a section in the discussion (lines 653-663), comparing the subunits detected in proteomic vs structural studies, both here and in our previous Toxoplasma complexome paper (PMID: 33651838). Overall, our complexome profiling experiments identify the majority of subunits seen in cryo-EM structures: 47 of 49 subunits of the Perkinsus mETC supercomplex structure, 30 out of 32 subunits from the Toxoplasma ATP synthase structure, and 33 out of 37 subunits from the Toxoplasma III-IV supercomplex structure.*

4. Identification of the ISR proteins as putative Rieske protein modifiers is interesting. The authors identify homologs in Toxoplasma and show mitochondrial localization for one of them. It would be helpful if the authors could discuss whether these “ISR” proteins were identified in their prior mitochondrial complexome study of Toxoplasma and give the fitness phenotype scores for these proteins in Toxoplasma based on prior CRISPR screens. Alternatively, or in addition, it would strengthen the impact of the present paper if the authors could use the tagged Toxoplasma line to show by BN-PAGE that the tagged ISR ortholog in Toxoplasma also migrates with Complex III.

*We agree that the role of TgISPR is an interesting avenue of investigation, and one that we are actively following up on.*

*To explore the impact of TgISPR, we have made conditional knockdown lines of this gene, shown in a figure below. No defect in Toxoplasma growth was observed by plaque assay. This is perhaps not surprising given a phenotype score from the genome-wide CRISPR screen (PMID: 27594426) of -0.64, indicating dispensability. By Native PAGE we also looked for comigration of ISPR with complex III in our overexpression line, but could not detect a clear signal (see below). We additionally made a conditional knockdown line of TgISPR in a background where the complex III subunit QCR7 was HA tagged. Depletion of TgISPR had no effect on complex III abundance or stability. Finally, TgISPR was not identified in our previous Toxoplasma complexome study. Together these results point to a limited role for ISPR in the tachyzoite life stage of Toxoplasma, on which these experiments were carried out.*

*Investigating further shows that the ISPR gene is more highly expressed in the bradyzoite life stage. This is show in 2 published transcriptome datasets (PMID: 31955846 and PMID: 30728393) and confirmed by us with qRT-PCR analysis (albeit with high variation) (see below). Our working hypothesis is that ISPR is a bradyzoite specific gene in Toxoplasma with a putative role in that stage. Exploring this requires extensive cell-biology and genetic studies in the challenging to work with bradyzoite. Between the long time that such work would take and our aim here, which is to report the composition of mitochondrial complexes, more work on ISPR is outside the scope of the current study. We have added additional text in the results section to explain this (lines 307-312, 317-320).*

*Figure for reviewers: Further characterisation of TgISPR (A) A conditional knockdown line of TgISPR was created using the ATc regulatable promoter replacement method (PMID: 22144892). Graph shows qRT-PCR analysis of transcript levels after addition of ATc, confirming downregulation of transcript. (B) Plaque assay of ISPR knockdown line in the presence and absence of ATc, which downregulates transcript levels. On the right is shown quantification of plaque size in parental and ISPR knockdown line. No defect is observed. (C) Native PAGE and immunoblot analysis of complex III subunit QCR7-HA, and ISPR overexpression line. Whole cell samples were run on the same gel, transferred to PVDF and probed with HA and Ty, respectively. No obvious band can be detected in the ISPR-OE-Ty line. (D) An ISPR knockdown line was created in the background of QCR7-HA. Samples were treated with ATc, to downregulate ISPR, and analysed by native-PAGE and immunoblot. A TOM40 SDS-PAGE and immunoblot was performed as a loading control. No defect in QCR7-HA signal is observed after downregulation of ISPR. (E) Tachyzoite and bradyzoite RNA samples were generated and qRT-PCR analysis performed to determine TgISPR transcript levels. TgISPR appears more highly expressed in bradyzoites.*

5. L314-316: In their discussion of PmATPTG1, can the authors comment if this protein sequence in Perkinsus, which appears to be encoded by a separate gene from cyt c1 (in contrast to Toxoplasma and Plasmodium), shows any sequence similarity (or not) to the leader sequence of the Perkinsus cyt c1 protein? Does this separation of genes suggest ancestral fusion or separation of these genes (ATPG1 and cyt c1) prior to evolutionary branching within Myzozoa? Did Perkinsus duplicate and diverge these genes (at least the leader sequence portions) or did Apicomplexa lose an ancestral, separate gene for ATPTG1?

*We could not detect any similarity between the leader sequence of CytC1 and ATPTG1 in Perkinsus (line 370). Our analysis in figure S4 suggest that the gene fusion between CytC1 and ATPTG1 is only found in a subset of apicomplexans- it is seen in all apicomplexans except cryptosporidium in our analysis. This suggests the gene fusion event occurred after the initial radiation of apicomplexans (shown by a green dot in figure S4) ie that this is a relatively late event happening once the apicomplexans have already split off from the other myzozoan lineages, rather than an event happening prior to evolutionary branching within Myzozoa. It isn’t possible to tell, from the data we have, the exact mechanism of the fusion event in Apicomplexa.*

6. Related to the above points, it would be helpful for the authors to provide a comprehensive table that displays the homologous proteins and differences found in P. marinus compared to P. falciparum and T. gondii. This table would help readers easily compare and contrast the complex components between apicomplexan parasites and P. marinus.

*We have added text and analysis comparing and contrasting mETC and ATP synthase composition in different lineages in response to other reviewer points. We have amended S4 table to show the correspomding experimentally validated subunits in Plasmdoium and Toxoplasma, and added a new supplementary table (s6), which allow comparison of homologous proteins between Perkinsus, Toxoplasma, Plasmodium and other species.*

*Other additions:*

*In addition to reviewer comments, we have made a few minor edits to improve grammar and readability. These are all highlighted in the “Revised manuscript with track changes” document.*

*Finally, we added some extra analysis of our proteomic data using amino acid sequences derived from the recent structure (PDB 9FQ7). These data were not publicly available when we performed our initial analysis. These extra subunits (SDHC, SDHH, QCR9, QCR11 and QCR 12) do not have a corresponding geneID in the current genome annotation (Uniprot UP000007800), but by including their amino acid sequence (derived from PDB 9FQ7) to our search database we detected 4 of them in our complexome dataset. This results in a more complete complexome dataset, and allows extra analysis of complex II subunit SDHC (lines 222-237, Fig S2B). We fell the addition of these new analysis strengthens the manuscript.*

*Response to journal requirements:*

*2) Author summary- a new author summary section has been included in the manuscript (lines 36-51).*

*4) Supplementary figures are now included in a separate file.*

*6) The proteomics dataset hosted on the massive server will of course be made freely accessible upon acceptance of the manuscript.*

*Additionally, we have a source data file of uncropped immunoblot and gel images used in the paper. Depending on journal policy, we can include this as supporting information, as a supplemental figure or on a separate online repository, as directed by journal staff.*
