# Supplementary material for: Complexome profiling of mitochondria in the myzozoan parasite of oysters, Perkinsus marinus
Source: PLoS Pathog. 2026 Sep 29;22(9):e1014543. doi: 10.1371/journal.ppat.1014543 (PMC13623125; doi:10.1371/journal.ppat.1014543)
Supplement: Peer review history

# Author comment: Author response to Decision Letter 0

**DOI:** 10.1371/journal.ppat.1014543.r001

**Transfer Alert**

This paper was transferred from another journal. As a result, its full editorial history (including decision letters, peer reviews and author responses) may not be present.

16 Mar 2026

# Peer review: Decision Letter 0

**DOI:** 10.1371/journal.ppat.1014543.r002

**Authors:** Margaret A Phillips, Michael L Reese

26 Apr 2026

Complexome profile of mitochondrial complexes in the myzozoan parasite of oysters, *Perkinsus marinus*

PLOS Pathogens

Dear Dr. Maclean,

Thank you for submitting your manuscript to PLOS Pathogens. After careful consideration, we feel that it has merit but does not fully meet PLOS Pathogens's publication criteria as it currently stands. Therefore, we invite you to submit a revised version of the manuscript that addresses the points raised during the review process.

Please submit your revised manuscript by Jun 25 2026 11:59PM. If you will need more time than this to complete your revisions, please reply to this message or contact the journal office at plospathogens@plos.org. When you're ready to submit your revision, log on to https://www.editorialmanager.com/ppathogens/ and select the 'Submissions Needing Revision' folder to locate your manuscript file.

Please include the following items when submitting your revised manuscript:

* A letter that responds to each point raised by the editor and reviewer(s). You should upload this letter as a separate file labeled 'Response to Reviewers'. This file does not need to include responses to any formatting updates and technical items listed in the 'Journal Requirements' section below.

* A marked-up copy of your manuscript that highlights changes made to the original version. You should upload this as a separate file labeled 'Revised Manuscript with Track Changes'.

* An unmarked version of your revised paper without tracked changes. You should upload this as a separate file labeled 'Manuscript'.

If you would like to make changes to your financial disclosure, competing interests statement, or data availability statement, please make these updates within the submission form at the time of resubmission. Guidelines for resubmitting your figure files are available below the reviewer comments at the end of this letter.

We look forward to receiving your revised manuscript.

Kind regards,

Michael L Reese, PhD

Academic Editor

PLOS Pathogens

Margaret Phillips

Section Editor

PLOS Pathogens

Editor-in-Chief

PLOS Pathogens

orcid.org/0000-0003-2946-9497

Editor-in-Chief

PLOS Pathogens

orcid.org/0000-0002-7699-2064

**Additional Editor Comments :**

Overall all three reviewers were quite positive about your manuscript. If you choose to resubmit, please respond productively to all comments. Reviewer #1 suggests that a more extensive phylogenetic analysis that includes comparison with other clades in SAR is warranted. They also suggest that the evolutionary analysis be more front-and-center and visually explained, as this is central to the paper. I agree with the reviewer that these changes would greatly improve the manuscript.

**Journal Requirements:**

1) We ask that a manuscript source file is provided at Revision. Please upload your manuscript file as a .doc, .docx, .rtf or .tex. If you are providing a .tex file, please upload it under the item type u2018LaTeX Source Fileu2019 and leave your .pdf version as the item type u2018Manuscriptu2019.

2) Please provide an Author Summary. This should appear in your manuscript between the Abstract (if applicable) and the Introduction, and should be 150-200 words long. The aim should be to make your findings accessible to a wide audience that includes both scientists and non-scientists. Sample summaries can be found on our website under Submission Guidelines:

https://journals.plos.org/plospathogens/s/submission-guidelines#loc-parts-of-a-submission

3) Please upload all main figures as separate Figure files in .tif or .eps format. For more information about how to convert and format your figure files please see our guidelines:

https://journals.plos.org/plospathogens/s/figures

4) We notice that your supplementary Figures are included in the manuscript file. Please remove them and upload them with the file type 'Supporting Information'. Please ensure that each Supporting Information file has a legend listed in the manuscript after the references list.

5) Please ensure that the funders and grant numbers match between the Financial Disclosure field and the Funding Information tab in your submission form. Note that the funders must be provided in the same order in both places as well. Currently, the order of this grant ( 23-06479X ) is different in both places

6) Thank you for stating that “Proteomics datasets have been deposited to the ProteomeXchange Consortium via the MassIVE partner repository with the dataset identifier MSV000100877." Please note that, though access restrictions are acceptable now, your entire data will need to be made freely accessible if your manuscript is accepted for publication. This policy applies to all data except where public deposition would breach compliance with the protocol approved by your research ethics board.

Note: If the reviewer comments include a recommendation to cite specific previously published works, please review and evaluate these publications to determine whether they are relevant and should be cited. There is no requirement to cite these works unless the editor has indicated otherwise.

**Reviewers' Comments:**

Reviewer's Responses to Questions

**Part I - Summary**

Please use this section to discuss strengths/weaknesses of study, novelty/significance, general execution and scholarship.

Reviewer #1: Myzozoans are a eukaryotic lineage that include apicomplexan parasites like Toxoplasma gondii, algae such as dinoflagellates, and the oyster parasite Perkinsus marinus. Numerous recent studies suggest that the composition of mitochondrial protein complexes from myzozoans differs considerably from other eukaryotic lineages. However, the bulk of these studies come from a couple of species of apicomplexans. In the study Maclean and colleagues provide a proteomic analysis of mitochondrial complexes in P. marinus. They identify numerous candidate protein complexes, and identify many proteins in these complexes that appear to be restricted to the myzozoan lineage. This fills important knowledge gaps around mitochondrial biology in a poorly studied lineage of the myzozoans, and provides greater clarity on the evolution of novel mitochondrial biology in the myzozoans generally. The study also provides an important starting point for understanding the mitochondrial biology of an important group of mollusc parasites. With the exception of the comparison of candidate ATP synthase subunits between Perkinsus and Toxoplasma (Figure 3), the study is largely a descriptive one. As noted in my major comment, the study would hold broader interest if the authors provided a more thorough comparison of the putative composition of mitochondrial complexes between Perkinsus and apicomplexans. Overall, the experiments described in the study are solid, convincing, clearly described, and lead to some interesting conclusions.

Reviewer #2: Maclean et al. report characterization of mitochondrial (and other cellular) protein complexes in Perkinsus parasites using native PAGE and tandem mass spectrometry. Prior work had identified expanded and divergent mitochondrial complex compositions in apicomplexan parasites, Toxoplasma and Plasmodium, relative to yeast and animals. The present manuscript set out to test and identify if Perkinsus parasites were similar to or different from Apicomplexa in their mitochondrial complexes and thus clarify if mitochondrial elaborations are unique to Apicomplexa or general to the broader clade of Myzozoa within which Perkinsus and Apicomplexa occupy distinct phyla.

The authors successfully profiled protein components of the major mitochondrial complexes involved in the respiratory chain, ribosomes, and protein import. Overall, complex compositions were generally similar to Toxo and Plasmodium, with a few differences. This observation strongly supports the conclusion that expanded mitochondrial complexes are a general evolutionary feature of Myzozoa that evolved prior to the divergence into distinct phyla. The quality of the work appears to be high. While the conclusions are not especially profound, the detailed study of parasitic relatives of major human pathogens will be a useful resource for comparative mitochondrial biology and its evolution among diverging eukaryotes.

Reviewer #3: In this study, MacLean et al. perform a complexome analysis in Perkinsus marinus, a protozoan parasite of oysters belonging to the myzozoa, a group that contains parasitic (apicomplexans) and free living (dinoflagellates) organisms. Using whole cell material and mitochondrially enriched fractions, the complexome analysis allowed the authors to identify several protein complexes within the mitochondrion of P. marinus. Among them, the complexes of the mitochondrial electron transport chain (mETC), the mitoribosome, the TOM complex, the proteasome and the endoplasmic reticulum membrane protein complex (EMC). Overall, the manuscript relies on the complexome analysis, which the group has extensive experience with—they have performed the same type of approach and analysis to another organism, the apicomplexan Toxoplasma gondii. In fact, they use their previously published data to compare the composition of the complexes between T. gondii and P. marinus. Comparing the composition of the complexes—particularly the complexes of the mETC—revealed that they are mostly conserved between the two organisms and likely evolved from a common ancestor. The paper provides important information on the overall conservation these complexes, while also highlighting potential differences and the potential presence of P. marinus—specific subunits, notably in its ATP synthase complex.

Complexome profile is a well suited approach to identify organellar protein complexes. The experiments are carefully executed, and this study really did a good job using both mitochondrially enriched samples and whole cell samples to determine the composition of the protein complexes. Good controls and markers to calibrate the sizes of the complexes (bovine mitochondrion membrane and NativeMark) were also used.

However, the manuscript is descriptive in nature. The results are limited to the comparison of the T. gondii and P. marinus complexes, and given that both organisms are myzozoans, a similar composition is not that surprising. I feel that the significance of these findings is currently limited because the authors do not move beyond those comparisons. The absence of functional validation represents a missed opportunity to substantiate the evolutionary hypotheses presented in the manuscript. While I think that performing experiments in P. marinus to validate their observations might be outside of the scope of the paper, the authors should carefully reword their discussion to highlight the limitations of their results.

**********

**Part II – Major Issues: Key Experiments Required for Acceptance**

Please use this section to detail the key new experiments or modifications of existing experiments that should be absolutely required to validate study conclusions.

Generally, there should be no more than 3 such required experiments or major modifications for a "Major Revision" recommendation. If more than 3 experiments are necessary to validate the study conclusions, then you are encouraged to recommend "Reject".

Reviewer #1: 1. A major conclusion of the study is that a lot of the unique features and proteins of apicomplexan mitochondrial complexes are conserved in Perkinsus. This provides evidence that these novelties were already present in the common ancestor of myzozoans. However, the study makes only a limited attempt at describing these orthologies, and buries a lot of it in the supplementary data (e.g. Table S4 and the ATPTG1 homology data). The study would be improved by providing a more thorough (and visual) analysis of which proteins identified in Perkinsus complexes have homologs in apicomplexans, and which of these proteins are present in the characterized complexes of apicomplexans. The authors might also consider providing a more detailed analysis of which Perkinsus proteins do NOT have homologues in apicomplexans (and vice versa), since this may point to lineage-specific novelties within the myzozoans (as they do for ATP synthase in Figure 3C). Additionally, it would be interesting to note which "novel" myzozoan mitochondrial complex proteins also have homologs in ciliates, given that ciliates are the sister taxon to the myzozoans.

Reviewer #2: 1. The current figures that predominantly show the BN-PAGE slices are not so helpful or informative in summarizing the key proteomic findings. Summary diagrams should be included as a panel within each figure to schematically depict the protein subunits of the studied complexes, similar to schematic figures included in the authors’ prior complexome studies of Toxoplasma (or something similar to Fig. 2 in PMID 23371551). Summary panels that list the identified subunits (perhaps in comparison to Toxoplasma and/or Plasmodium) should also be considered. If necessary, some of the slice details in the present figures can be decanted to supporting figures.

2. Evolutionary and phylogenetic comparisons are central to the major conclusions of this manuscript. It would be helpful to include a simple phylogenetic tree as a new Figure 1, showing the relationships between Perkinsus, Apicomplexa (including Toxo and Plasmodium that are key comparison organisms), and other major eukaryotic clades (including opisthokonts).

Reviewer #3: Line 49: Do we know which subunits of the mETC complexes are encoded in the mitochondrial genome of P. marinus, and are those the same as the ones in apicomplexans? If so, the authors should mention it.

Line 110: The last sentence of this paragraph is somewhat misleading.

Although the authors convincingly show that their mitochondrially enriched fractions are indeed enriched with mitochondria, the ER is also present. Please mention that.

On figure 2E, What is the degree of confidence in the alphafold predictions of ISPR? This is also valid for the other proteins shown in the paper.

Line 167: When the authors are comparing their mitochondrial datasets with published ones from Toxoplasma and Plasmodium, what determines a “sufficient” coverage? Is there a certain percentage that needs to be attained? Is this arbitrary? The authors should explain their reasoning.

Line 210: has the homolog of BCS1 been observed in the author’s published T. gondii complexome analysis? If so they should mention it, it not they should also mention it and explain why is that.

Line 305: Since a putative complex III assembly factor was detected, could those proteins be ATP synthase assembly factors instead of subunits of the complex? Is there a way to distinguish those using the author’s approach?

Line 413-414: Is there any relevance of detecting proteasome proteins in the mitochondrially enriched fractions? Could this be a contamination? The authors did not mention this in the manuscript (Table S5).

Why did the authors used BDDM instead of digitonin as they have previously reported (PMID: 33651838)? In BN-PAGE, digitonin seem to reveal complex arrangements that are lost with BDDM. A comparison of using both detergents will be useful.

**********

**Part III – Minor Issues: Editorial and Data Presentation Modifications**

Please use this section for editorial suggestions as well as relatively minor modifications of existing data that would enhance clarity.

Reviewer #1: 2. Line 75. “While apicomplexan mitochondrial genomes encode the lowest number of protein-coding genes known [20], their ribosomal RNAs (rRNAs) are uniquely extremely fragmented [21,22].” True that mitochondrial rRNAs are extremely fragmented in apicomplexans, but this isn’t entirely unique (e.g. also occurs in dinoflagellates and some ciliates). Additionally, Chromera velia encodes two protein-coding genes in its mitochondrial genome, which is fewer than in apicomplexans. Consider re-wording for accuracy.

3. Line 173. “recent studies in apicomplexans suggest a much-expanded subunit repertoire compared to the commonly studied opisthokont models”. Given that opisthokont protein complex compositions provide an important comparison to the myzozoan complexes throughout the manuscript, consider briefly mentioning here which lineages belong to the opisthokonts.

4. Line 194. “This size of complex II is consistent with the BN-PAGE in-gel stain (Fig 1B).” Given that the in-gel stain depends on enzymatic activity, it might be worth pointing out this size is therefore likely the enzymatically active version of the complex.

5. Line 210. “we observed Pmar_PMAR021073, a predicted homolog of the complex III assembly factor BCS1, with a peak in the gel slice below mature complex III, at ~820 kDa … suggesting that in our mitochondrially-enriched complexome, we observe a late assembly intermediate as well as mature complex III. ” This claim seems a bit speculative, since there are other proteins in the same ~820 kDa peak that are presumably not part of any assembly intermediate.

6. Line 233. “supercomplexes” (spelling)

7. Line 240. “complex IV subunits have peaks at ~760-840, ~1,300-1,400 and ~2,800 kDa”. How were these mass estimates determined? If none of the calibration standards are above 2,800 kDa, then probably better to describe this without putting a number on the mass.

8. Line 245. “demonstrating that the enlarged mETC complexes observed in Apicomplexa are conserved across a broader group within the myzozoans and thus likely evolved in their common ancestor.” The masses of the complexes being enlarged is not, in itself, strong evidence that complex enlargement occurred in the common ancestor of myzozoans. Rather, the presence of myzozoan lineage-specific subunits (which are contributing to the larger masses) suggest that the novelty observed in myzozoan mitochondrial complexes was already present in the common ancestor of the lineage. A more thorough comparison of orthology in mitochondrial complex proteins across myzozoans may be a more robust way to phrase this argument (see Major comment 1).

9. Line 312. “Homology analysis showed that this fusion was conserved across a subset of apicomplexans and that outside this group ATPTG1 and CytC1 are encoded by separate genes [10] (S3C, D, S4 Figs, Table S7).” The authors could be clearer that they encoded on separate genes in Perkinsus. Similarly, in the next line, the authors could be clearer that they are referring to the P. marinus homolog of ATPTG10: “Our detection of ATPTG1 in complex with ATP synthase [in P. marinus] strongly suggests …”

10. Table S4. Consider adding a comparison to additional apicomplexan species (especially Plasmodium falciparum, given that complexome data exist for that organism), and also listing VEuPathDB gene IDs for the equivalent apicomplexan homologs. See also Major comment 1 – consider providing a more visual representation of these data, and including at least some of the analysis in the main figures.

11. Line 349. “11 Toxoplasma proteins interact each with multiple rRNAs and therefore were assigned a putative role as “joints”.” Joints in what sense? That they are joining different rRNA fragments? This could be clearer.

12. Line 400. “Further investigations will be required to identify if additional TOM subunits exist, in particular divergent versions of the receptors Tom20 and Tom70.” Structural analyses of the TOM complex of opisthokonts have characterized a so-called “core” TOM complex, consisting of Tom40/22/7/6/5 (e.g. PMIDs 31740857 and 33083003). This suggests that the receptor proteins of this complex (e.g. Tom70/20 in opisthokonts) may associate only transiently with the complex. The identification of candidate Tom5 and Tom6 homologs in Perkinsus suggest that the entire core TOM complex must have evolved very early in eukaryotic evolution, which is something the authors could consider highlighting. I was curious whether the candidate Perkinsus Tom5/6 homologs are present in apicomplexans as well? This may be worth commenting on here.

13. Line 415. “we searched our mitochondrial complexome for the endoplasmic reticulum membrane protein complex (EMC).” What is the role of the EMC in ER biology? What is known about its presence in different eukaryotic lineages?

14. Line 448. “… findings have been found …” Consider re-wording for clarity.

15. Figure 1 legend. Define “CBB”

Reviewer #2: 1. The current title seems redundant. The authors may wish to consider a revised title such as “Molecular profiling of mitochondrial complexes in…”.

2. L81-83: Can the authors elaborate on this point? How many of the identified subunits of the studied complexes were predictable by sequence homology? How many would not have been predictable from sequence comparisons alone? The authors invested substantial effort to experimentally identify these subunits. It would be helpful in the Discussion to comment on the role and impact of experiment in an era in which sequence comparisons (now boosted by the ability to perform structure-based similarity searches as well) are increasingly facile and powerful.

3. Related, several of the authors of the present manuscript are also listed as authors on a preprint structural study of Perkinsus mitochondrial supercomplexes available at bioRxiv. A more detailed comparison of the compositions of mitochondrial complexes determined by mass spectrometry in this study versus direct structural visualization in the parallel study would be very helpful, either within Results or a summary Discussion. The present study reported analysis of additional complexes not observed in the structural paper, but comparison between those complexes in common between the two studies would be appropriate.

4. Identification of the ISR proteins as putative Rieske protein modifiers is interesting. The authors identify homologs in Toxoplasma and show mitochondrial localization for one of them. It would be helpful if the authors could discuss whether these “ISR” proteins were identified in their prior mitochondrial complexome study of Toxoplasma and give the fitness phenotype scores for these proteins in Toxoplasma based on prior CRISPR screens. Alternatively, or in addition, it would strengthen the impact of the present paper if the authors could use the tagged Toxoplasma line to show by BN-PAGE that the tagged ISR ortholog in Toxoplasma also migrates with Complex III.

5. L314-316: In their discussion of PmATPTG1, can the authors comment if this protein sequence in Perkinsus, which appears to be encoded by a separate gene from cyt c1 (in contrast to Toxoplasma and Plasmodium), shows any sequence similarity (or not) to the leader sequence of the Perkinsus cyt c1 protein? Does this separation of genes suggest ancestral fusion or separation of these genes (ATPG1 and cyt c1) prior to evolutionary branching within Myzozoa? Did Perkinsus duplicate and diverge these genes (at least the leader sequence portions) or did Apicomplexa lose an ancestral, separate gene for ATPTG1?

6. Related to the above points, it would be helpful for the authors to provide a comprehensive table that displays the homologous proteins and differences found in P. marinus compared to P. falciparum and T. gondii. This table would help readers easily compare and contrast the complex components between apicomplexan parasites and P. marinus.

Reviewer #3: None

**********

PLOS authors have the option to publish the peer review history of their article (what does this mean?). If published, this will include your full peer review and any attached files.

If you choose “no”, your identity will remain anonymous but your review may still be made public.

**Do you want your identity to be public for this peer review?**  For information about this choice, including consent withdrawal, please see our Privacy Policy.

Reviewer #1: No

Reviewer #2: No

Reviewer #3: No

[NOTE: If reviewer comments were submitted as an attachment file, they will be attached to this email and accessible via the submission site. Please log into your account, locate the manuscript record, and check for the action link "View Attachments". If this link does not appear, there are no attachment files.]

**Figure resubmission:**

**Reproducibility:**

?>

# Author comment: Author response to Decision Letter 1

**DOI:** 10.1371/journal.ppat.1014543.r003

15 Jul 2026

Attachment Submitted filename: Response to reviewers.docx

# Peer review: Decision Letter 1

**DOI:** 10.1371/journal.ppat.1014543.r004

**Authors:** Margaret A Phillips, Michael L Reese

11 Aug 2026

Dear Dr Maclean,

We are pleased to inform you that your manuscript 'Complexome profile of mitochondrial complexes in the myzozoan parasite of oysters, *Perkinsus marinus* ' has been provisionally accepted for publication in PLOS Pathogens.

Before your manuscript can be formally accepted you will need to complete some formatting changes, which you will receive in a follow up email. A member of our team will be in touch with a set of requests.

Please note that your manuscript will not be scheduled for publication until you have made the required changes, so a swift response is appreciated.

IMPORTANT: The editorial review process is now complete. PLOS will only permit corrections to spelling, formatting or significant scientific errors from this point onwards. Requests for major changes, or any which affect the scientific understanding of your work, will cause delays to the publication date of your manuscript.

Should you, your institution's press office or the journal office choose to press release your paper, you will automatically be opted out of early publication. We ask that you notify us now if you or your institution is planning to press release the article. All press must be co-ordinated with PLOS.

Thank you again for supporting Open Access publishing; we are looking forward to publishing your work in PLOS Pathogens.

Best regards,

Michael L Reese, PhD

Academic Editor

PLOS Pathogens

Margaret Phillips

Section Editor

PLOS Pathogens

Sumita Bhaduri-McIntosh

Editor-in-Chief

PLOS Pathogens

orcid.org/0000-0003-2946-9497

Michael Malim

Editor-in-Chief

PLOS Pathogens

orcid.org/0000-0002-7699-2064

***********************************************************

As you see all three reviewers are satisfied with the manuscript. Each has some suggestions for clarity, which you may consider updating, though I do not feel the paper requires another round of review.

Reviewer Comments (if any, and for reference):

Reviewer's Responses to Questions

**Part I - Summary**

Please use this section to discuss strengths/weaknesses of study, novelty/significance, general execution and scholarship.

Reviewer #1: In their revision, Maclean and colleagues have done an excellent job in placing their findings into a broader evolutionary context. This addresses my major criticism from the original manuscript. I also really liked the authors arguments about the relevance of lab-based experiments that is now included in the discussion (from Line 549). I congratulate the authors on an excellent and well written study. I have a few minor comments that the authors may consider addressing before publication.

Reviewer #2: The authors have thoughtfully and in most cases strongly addressed the prior reviewer critiques. This manuscript will be a valuable resource for comparative mitochondrial biology between myzozoan organisms and raises several interesting evolutionary questions for future studies. I have a few minor and optional considerations for the authors, noted below.

Reviewer #3: I believe the revised version of the paper addressed all of the reviewers' comments. This version has been strengthened and, aside from a few minor changes that I think will increase the clarity of the manuscript, is ready for publication. Congratulations to all the authors.

**********

**Part II – Major Issues: Key Experiments Required for Acceptance**

Please use this section to detail the key new experiments or modifications of existing experiments that should be absolutely required to validate study conclusions.

Generally, there should be no more than 3 such required experiments or major modifications for a "Major Revision" recommendation. If more than 3 experiments are necessary to validate the study conclusions, then you are encouraged to recommend "Reject".

Reviewer #1: None

Reviewer #2: None

Reviewer #3: (No Response)

**********

**Part III – Minor Issues: Editorial and Data Presentation Modifications**

Please use this section for editorial suggestions as well as relatively minor modifications of existing data that would enhance clarity.

Reviewer #1: Line 175 (with similar comments applying to line 177, 182 and 186). “Our datasets contained putative Perkinsus homologs of between 65-67% of the Toxoplasma proteins found in the mitochondrial clusters.” I don’t understand how the 65-67% range was determined. Presumably different alogrithms (the BLASTP and HMM searches described in the methods perhaps?) gave differing results. Or is this reporting values for the two replicates? This could be explained briefly.

Line 215. “The whole-cell complexome dataset also revealed evidence of a sub-assembly containing SDHA and SDHB migrating at ~320-350 kDa (S2A Fig).” But, curiously, a depletion of SDHA and SDHB in the larger ~1 MDa complex with the other putative SDH subunits. The authors could note this. Perhaps the whole-cell preparation led to partial breakdown of this complex?

Line 489. “Since immunoblot analysis suggested co-purification of the endoplasmic reticulum” – consider referencing the relevant figure here.

Reviewer #2: 1. The redundant title still seems sub-optimal. How about “Mitochondrial complexome profiling in the myzozoan parasite of oysters, Perkinsus marinus”? Or “Complexome profiling of mitochondria in the myzozoan parasite of oysters, Perkinsus marinus”. The authors should decide what they like best and go with that title.

2. In the new Figure 1, an alternative oxidase is depicted in the Perkinsus ETC diagram but is not mentioned in the text. Does Perkinsus encode a putative AOX and did the authors detect it in their study? That observation seems worth noting briefly in the manuscript somewhere, and the authors might add the presence or absence of AOX to Fig. 1B as a further differentiation between these organisms.

3. Identification of BCS1 as part of a mito complex seems interesting and worth noting. Rather than removing mention of identifying BCS1, I suggest just doing a very brief analysis of what other proteins co-migrate in that slice and suggesting multiple possible models/interpretations of what it could represent. I’m not aware of any coherent precedent in yeast or humans for BCS1 forming part of a larger complex. Even if the significance of this observation isn’t definitive, I would rather see it still mentioned than removed entirely, as it could seed or be relevant for future studies.

Reviewer #3: Figure 2B is confusing. I had to go back and forth the legend/manuscript to remind myself what each slice corresponded to. The authors should just explicitly label each gel slice with the corresponding complex they are trying to observe. I would suggest add that at the top of the figure, then at the bottom you can add the type of assay used for each slice, no need to indicate the gel and detergent, that can be done in the legend text.

Line 178 – 188: I appreciate that the authors compared their complexome datasets with readily available hyperLOPIT data from Toxoplasma gondii and Plasmodium falciparum. However, I would like to see a table/figure reflecting the percentages mentioned the text, that would be highly informative.

For each one of the figures representing results from complexome analysis, it would be great if the authors could highlight (in red, in bold, etc.) the proteins that they focus on in the text: for instance, SDHA and SDHB in Figure 2C, ATPPM1 and 2 in Figure 3A, and mS130, mL143, mL153, and mL161 in Figure 4. Highlighting them will make them instantly easier to find among the other numerous subunits of each complex.

**********

PLOS authors have the option to publish the peer review history of their article (what does this mean?). If published, this will include your full peer review and any attached files.

If you choose “no”, your identity will remain anonymous but your review may still be made public.

**Do you want your identity to be public for this peer review?**  For information about this choice, including consent withdrawal, please see our Privacy Policy.

Reviewer #1: No

Reviewer #2: No

Reviewer #3: No

# Editor report: Acceptance letter

**DOI:** 10.1371/journal.ppat.1014543.r005

**Authors:** Margaret A Phillips, Michael L Reese

Dear Dr Maclean,

We are delighted to inform you that your manuscript, "Complexome profile of mitochondrial complexes in the myzozoan parasite of oysters, *Perkinsus marinus* ," has been formally accepted for publication in PLOS Pathogens.

We have now passed your article onto the PLOS Production Department who will complete the rest of the pre-publication process. All authors will receive a confirmation email upon publication.

Thank you again for supporting open-access publishing; we are looking forward to publishing your work in PLOS Pathogens.

Best regards,

Dominique Soldati-Favre

Editor-in-Chief

PLOS Pathogens
